# Evolution of H3N2 Influenza Virus in a Guinea Pig Model

**DOI:** 10.1371/journal.pone.0020130

**Published:** 2011-07-22

**Authors:** Jinxue Long, Ruth V. Bushnell, John K. Tobin, Keyao Pan, Michael W. Deem, Peter L. Nara, Gregory J. Tobin

**Affiliations:** 1 Biological Mimetics, Inc., Frederick, Maryland, United States of America; 2 Department of Bioengineering and Physics and Astronomy, Rice University, Houston, Texas, United States of America; 3 Department of Biological Sciences, College of Veterinary Medicine, Iowa State University, Ames, Iowa, United States of America; Johns Hopkins University, United States of America

## Abstract

Studies of influenza virus evolution under controlled experimental conditions can provide a better understanding of the consequences of evolutionary processes with and without immunological pressure. Characterization of evolved strains assists in the development of predictive algorithms for both the selection of subtypes represented in the seasonal influenza vaccine and the design of novel immune refocused vaccines. To obtain data on the evolution of influenza in a controlled setting, naïve and immunized Guinea pigs were infected with influenza A/Wyoming/2003 (H3N2). Virus progeny from nasal wash samples were assessed for variation in the dominant and other epitopes by sequencing the hemagglutinin (HA) gene to quantify evolutionary changes. Viral RNA from the nasal washes from infection of naïve and immune animals contained 6% and 24.5% HA variant sequences, respectively. Analysis of mutations relative to antigenic epitopes indicated that adaptive immunity played a key role in virus evolution. HA mutations in immunized animals were associated with loss of glycosylation and changes in charge and hydrophobicity in and near residues within known epitopes. Four regions of HA-1 (75–85, 125–135, 165–170, 225–230) contained residues of highest variability. These sites are adjacent to or within known epitopes and appear to play an important role in antigenic variation. Recognition of the role of these sites during evolution will lead to a better understanding of the nature of evolution which help in the prediction of future strains for selection of seasonal vaccines and the design of novel vaccines intended to stimulated broadened cross-reactive protection to conserved sites outside of dominant epitopes.

## Introduction

Globally, influenza is responsible for 250,000 to 500,000 deaths annually and is considered one of the most important respiratory pathogens of humans [1], [2], [3]. In the majority of the past ten years, H3N2 has dominated in prevalence of infection and disease over H1N1, H2N2, and influenza B. In the United States alone, approximately 5–20% of the population contracts influenza illnesses leading to about 240,000 hospitalizations and 40,000 deaths with the majority due to H3N2 [4], [5]. In addition to morbidity and mortality, influenza causes an annual economic impact in the range of $80B in this country alone [5]. Although vaccination is one of the most important preventative methods, the current vaccine design is far from perfect. Due to the antigenic evolution of the virus and strain-specific immune responses of the host, the vaccine requires reformulation every year or two to offer significant protection against circulating strains not represented in the vaccine. In the 2007–08 seasons, for example, the vaccine was composed of viruses antigenically similar to A/Solomon Islands (H1), A/Wisconsin (H3) and B/Malaysia (Victoria). According to the results of antigenic surveillance done by CDC, 91% of the H1N1 viruses circulating in 2007–8 were similar to the vaccine strain, but only 29% of the H3N2 strains were characterized as A/Wisconsin-like virus. The vaccine was not a good match against circulating strains in 2007–8, causing larger than normal numbers morbidity and mortality predominantly due to Brisbane/2007 - like viruses. In an effort to match the newly emerged dominant virus strain, the Brisbane/2007 was then chosen to be the H3N2 component for the 2008–9 and 2009–2010 Northern Hemisphere vaccines. Due to the uncertainty in the composition of future evolved strains, there are no guarantees that the subtype selected for a vaccine will be a close enough match against future strains that emerge from antigenic drift. Thus, improvements in predictive capabilities could lead to more effective vaccines.

The majority of the efforts expended to predict seasonal circulating influenza strains and the subsequent selection of the most appropriate vaccine strains are performed on an uncontrolled background of accumulated influenza immunity and viral evolution in the human host. The use of human natural infection data, rather than viral evolution data derived from well-controlled animal studies confounds the interpretation of both the serological and sequence data. The presence of various serologically cross-reactivate strains and subtypes of the virus along with residual host cross-reactivity due to prior infection and vaccination from previous years also adds layers of complexity to the interpretation of serological and virological data [6]. This loss of specificity of the recall immune response to some strains imparts immune selection in ways that are not fully understood when it comes to the immunodominant HA epitopes found on the virus. Thus, it would appear useful to derive more in-depth understandings of influenza evolution in more controlled experimental and immune settings, so as to augment the predictive power of those tasked with choosing the composition of seasonal vaccine strains. Furthermore, novel technologies, such as immune refocusing, which utilizes information about the immunodominance of the antigenic sites for their removal and have succeeded in preclinical studies of inducing enhanced cross-reactive immunity would benefit from such data [7]. Thus, we studied the evolution of the hemagglutinin (HA) protein of a selected H3N2 seasonal strain of virus in the Guinea pig animal model [8].

Influenza viruses have negative-strand segmented genomes that are replicated by a viral polymerase that has a high error rate [9], [10], [11]. The viral polymerase subunits PB1, PB2, PA, and NP form complexes that replicate the viral RNA strands with error rates in the range of 10^−4^ to 10^−5^ misincorporations per nucleotide per round of RNA synthesis [10], [11]. Although the majority of variants produced by the high mutation rate are expected to possess reduced viability, the availability of a pool of variants facilitates rapid evolution. Changes in amino acids located in immunodominant epitopes foster escape from immune pressure. Misincorporations by the replicase have been thought to occur randomly throughout the genome and supply the pool of variants from which evolutionarily fit strains develop. Therefore, highly variant domains are likely signs of immunological selection.

Several animal models have been used to study influenza infection and immunity. Although mouse, rat, and ferret models dominate the recent literature [8], [12], [13], [14], [15], publications from Lowen et al and Bushnell et al have advanced the Guinea pig as a relevant model of human influenza virus infection, transmission, and serology [8], [16]. Guinea pigs are susceptible to infection by unadapted human influenza virus strains and can transmit the virus to cage mates via aerosol [8]. The infection is largely centered in the upper respiratory tract and produces little outward signs of disease. In the present study, virus progeny recovered from nasal washes were examined for HA sequence variations. The data show a non-random propensity for mutations in the major antigenic sites. The present study examines the variations in the HA of A/Wyoming/2003 (H3N2) that occur in both naïve and immune animals for the dual purposes of assessing the model for recapitulation of virus evolution in humans and identifying evolutionary trends which can be used for prediction of future strains and subsequent design of novel vaccine candidates.

## Results

### Immunization and infection of Guinea pigs

Guinea pigs were infected with influenza A/Wyoming/03/2003 prior to or after immunization with homologous purified HA ectodomain protein. Theories on the mechanism by which antigenic drift occurs in vivo are based largely on studies of the HA sequence of natural variants and of mutants that escape *in vitro* selection by anti-HA antibodies possessing virus neutralizing activities [17]. Numerous reports have indicated that the HA protein can mediate immunity from infection or disease and suggest that the HA protein could become a viable subunit vaccine candidate.

**Figure 1:**
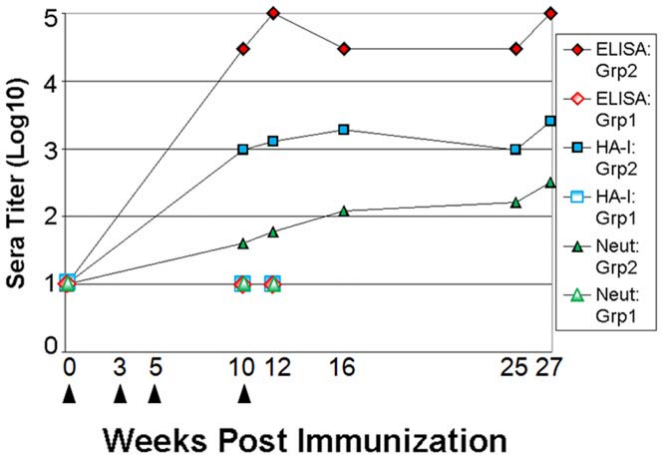
Immunological analysis of Guinea pig serum pools. Animals in Group 1 were inoculated with a mock preparation of antigen (open symbols) and then infected with homologous influenza A/Wyoming/03/2003. Animals in Group 2 were immunized with recombinant HA compounded in Incomplete and Complete Freund's adjuvant (solid symbols) prior to infection. Serum pools were assayed for antigen binding antibodies by ELISA against commercially prepared HA glycoprotein (diamond). Antiviral activities were measured by both hemagglutinin inhibition (square) and virus neutralization (triangle) assays. Arrows underneath graph indicate dates of inoculation. Immunized Guinea pigs were challenged with influenza virus on Week 25.

Selected groups of Guinea pigs were immunized by subcutaneous injection of purified recombinant HA ectodomain protein as described in Materials and Methods. Anti-HA antibody serum titers were followed to assess the immune status of the animals and to quantitate immunological pressure against the HA glycoprotein. Antigen-binding titers were measured by ELISA against commercially produced HA (Protein Sciences, Inc.) and antiviral activities were assessed by both hemagglutinin inhibition (HI) and virus neutralization (VN) assays. Figure 1 demonstrates during the course of the immunization phase for experimentally immunized Group 2 animals, ELISA titers rose to 1∶100,000, HI to 1∶ 2564, and VN titers to 1∶160. Sera from Guinea pigs in the mock immunized, naive group (Group 1) did not increase in titer from a baseline value of 1∶10 for all three assays. After 25 weeks, the Guinea pigs were inoculated intranasally with 10^4^ plaque forming units (PFU) of virus stock Wy-B4 produced from a plaque-purified and sequenced isolate of the reassorted virus, A/Wyoming/03 X PR8 X161B. The serological titers showed a slight increase after inoculation with virus (week 25 to 27).

### HA sequence data from progeny virus

The HA glycoprotein mediates virus-cell interactions and is the antigenically dominant surface protein. During virus maturation, the HA0 protein encoding by the HA gene is cleaved into HA1 and HA2 subunits. HA1 contains five defined epitopes involved in immunity and the mutations occurring in HA1 play key roles in virus escape from host immune pressure. A fragment (nt. 1–1,100) of the influenza RNA segment 4 encoding the HA1 subunit and all of the recognized HA epitopes was sequenced for the evolutionary study.

Nasal washes collected 3 days post-infection (p.i.) were titrated on MDCK cell monolayers to determine virus concentration. Viral RNA was purified directly from nasal washes, reverse transcribed, and used to produce molecular clones of the HA gene fragment for sequencing. 514 independently derived plasmid clones from naïve animals and 210 independently derived plasmid clones from immunized animals were sequenced and compared to the original sequence of the Wy-B4 HA gene. Table 1 contains a summary of the sequence data. Complete lists of the mutated strains are presented in Tables S1 and S2. Mean virus titers in nasal washes of HA-immunized animals were reduced approximately 10-fold in comparison to titers from naïve guinea pigs (10^3^ vs 10^4^ pfu/mL). 6% of the plasmid clones sequenced from naïve and 24.3% of the clones from immunized animals contained at least one mutation in the sequenced HA.

**Table 1:** Sequence analysis of progeny virus from influenza infection of guinea pigs.

Animal group	Immune status	Nasal titers (pfu/ml)	Sequenced HA-1 genes	HA variant strains	Total	Synonymous mutations	Non-synon. mutations
Group 1	Naïve	104	514	31(6.0%)	66	27(40.9%)	39(59.1%)
Group 2	immunized	103	210	51(24.3%)	157	94(59.9%)	63(40.1%)
Statistical				P<0.01	Statistical	P<0.01	P<0.01
significance				p = 2.017×10^−12^	significance	p = 9.5×10^−3^	p = 9.5×10^−3^
(X2)				(Significant)	(X2)	(Significant)	(Significant)

Analysis of HA variants showed that of the mutations found in naïve animals, 40.9% (27/66) did not affect amino acid changes (synonymous) while 59.1% (39/66) caused an amino acid substitution or deletion (non-synonymous). Conversely, mutations from immune guinea pigs included 59.9% (94/157) synonymous and 40.1% (63/157) non-synonymous changes (see table 1). Interestingly, the average number of mutations per variant found in naïve animals was 2.1 compared to 3.1 RNA mutations per variant strain in immunized animals. The amino acid mutation rate was calculated using the total number of non-synonymous mutations observed per amino acid residue. The HA-1 mutation rates for non-synonymous mutations in naïve and immune animals were calculated to be 2.3×10^−4^ and 9.0×10^−4^ residues mutated per amino acid, respectively.

### Analysis of mutations for changes in charge and hydrophobicity

The type of amino acid mutations that occur may either facilitate or hinder antigenic escape and replicative fitness. Sequence analysis of evolved influenza H3N2 HA from human samples indicates that changes in charge or hydrophobicity properties of the substituted amino acids in epitopes may be a major driver of antigenic escape [18]. Therefore, the physical properties of the mutations observed in progeny virus were analyzed to determine whether such correlations can be found in the variants observed in the progeny virus from Guinea pig infections and, if so, whether the correlations reflect immune status. Examination of mutations across the entire HA-1 regions showed that a slightly higher percentage of the variants from immunized animals involved charge changes compared to virus progeny from naïve animals (Table 2, 55.6% vs. 48.7%). Interestingly, a higher percentage of variants from naïve animals had changes in the hydrophobicity state as compared to variants from immunized Guinea pigs (33.3% vs. 11.1%). A Pearson Chi-squared statistical analysis of these data suggests that the differences in the percentage of charge changes between the two groups are not significant (p>0.05) but differences between hydrophobic states are significant (p<0.01).

**Table 2:** Analysis of amino acids in HA variants.

Animal Group	Immune Status	Total Variants	Charge Changes	Hydrophobicity Changes
1	Naïve	39	19 (48.7%)	13 (33.3%)
2	Immunized	63	35 (55.6%)	7 (11.1%)
Statistical			P>0.05	P<0.01
significance			p = 0.537	p = 0.006013
(X2)			(Not Significant)	(Significant)

### Frequency of mutations by position

**Figure 2:**
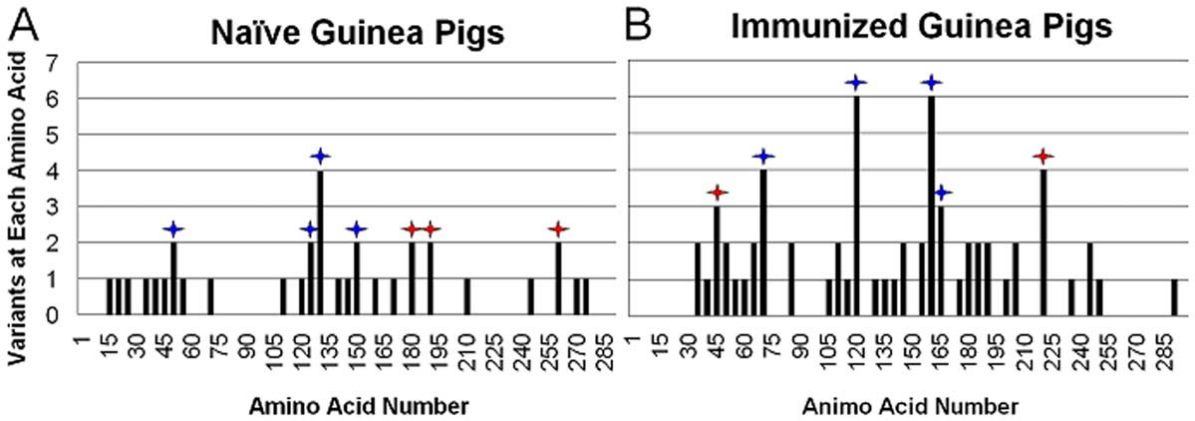
Histogram of influenza mutations by HA amino acid residue. Bars show the number of mutations at each amino acid observed in variants isolated from nasal washes of influenza virus infected Guinea pigs by sets of five residues as numbered. Panel A: variants from naïve Guinea pigs, Panel B: variants from HA-immunized animals. Blue stars above bars indicate mutations that occurred within known epitopes. Red stars above bars indicate mutations that occurred outside known epitopes.

**Figure 3:**
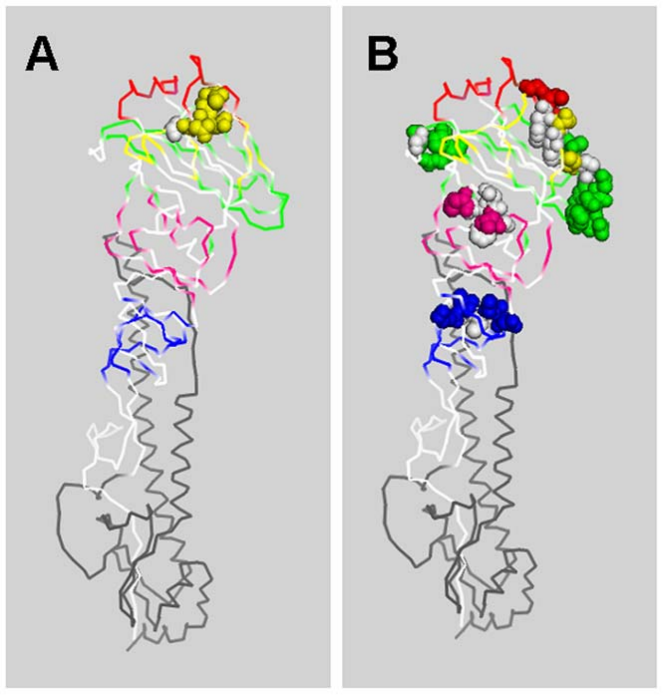
Location of most frequently occurring variants on 3D structure. Amino acids in the epitopes of the ribbon diagram of the HA protein from the related H3N2 strain X31 was colorized such that Epitope A = Yellow, B = Red, C = Blue, D = Green, and E = Pink. Amino acid residues with the highest frequency of variation are shown as balls. Panel A shows mutations from naïve Guinea pigs and Panel B from immunized animals. Figure drawn using PyMOL from 2VIR.pdb [19].

With the aim of identifying “hot spots” of variation, the position of the mutations in the variants were tabulated by groups of 5 amino acids and graphed (Figure 2). As expected, the mutations were not evenly scattered across the HA gene in a random pattern. In the naïve group, the distribution of variants in the virus progeny contained 18 sites with one variant, 6 sites with two, one site with four, and 327 (92.9%) sites without variants. The distribution of variants in the progeny from immunized animals showed 13 sites with one variant, 12 sites with two, two sites each with either three, four, or six, and 323 (91.5%) sites without variants. The Graphs in Figure 2 indicate that in the progeny virus from immunized animals there were four “hot spots” with a frequency of at least 4 or more mutations. The observation that multiple strains contained variants in these positions suggest that immune pressures are the highest at these sites. The region from 75–80 region had mutations Q75R, C76R, D77G and D77N; 125–130 had two at N126D and four at G129E; 165–170 had one N165G, one N165S, two T167A, two P169L; and region 225–230 had one I226V, one R228D and two R229D (See also Tables S1 and S2 for complete list of variants). The regions of the highest frequency of variations (hot spots) have been localized on crystallographic structures of the HA ectodomain using the related structure from H3N2 strain X31 (Figure 3, adapted from 2VIR.pdb, [19]). In Figure 3, epitopes are colorized and the hot spots of variation shown as balls for progeny virus from naïve (Panel A) and immunized Guinea pigs (Panel B). The figure shows that the majority of the hot spots are on the surface of the HA molecule and in epitope regions.

### Association of mutations with epitopes

A growing body of published data from the analysis of human viruses indicates that the process of influenza is not random and that the majority of selected variability occurs in the epitopes. The location of the five major epitopes of the HA glycoprotein has been determined [6], [20]. The effect of immunological pressure on the relative ratios of mutations accumulated in infected Guinea pigs within and outside the five defined epitopes was examined (Figure 3). Variants isolated from immunized animals had a higher percentage of mutations within the defined epitopes as compared to naïve Guinea pigs (38.5% vs. 63.5%). In addition, the types of amino acid changes found in the variants were also different in progeny virus sampled from immunized and naïve animals. Overall, the fraction of mutations that altered the charge of the residue was 65.7% and 42% in immunized and naive Guinea pigs respectively. Furthermore, hydrophobicity was affected by 71.4% and 38.5% of the mutations, in immunized and naïve animals.

### Analysis of mutants for changes in putative glycosylation

**Figure 4:**
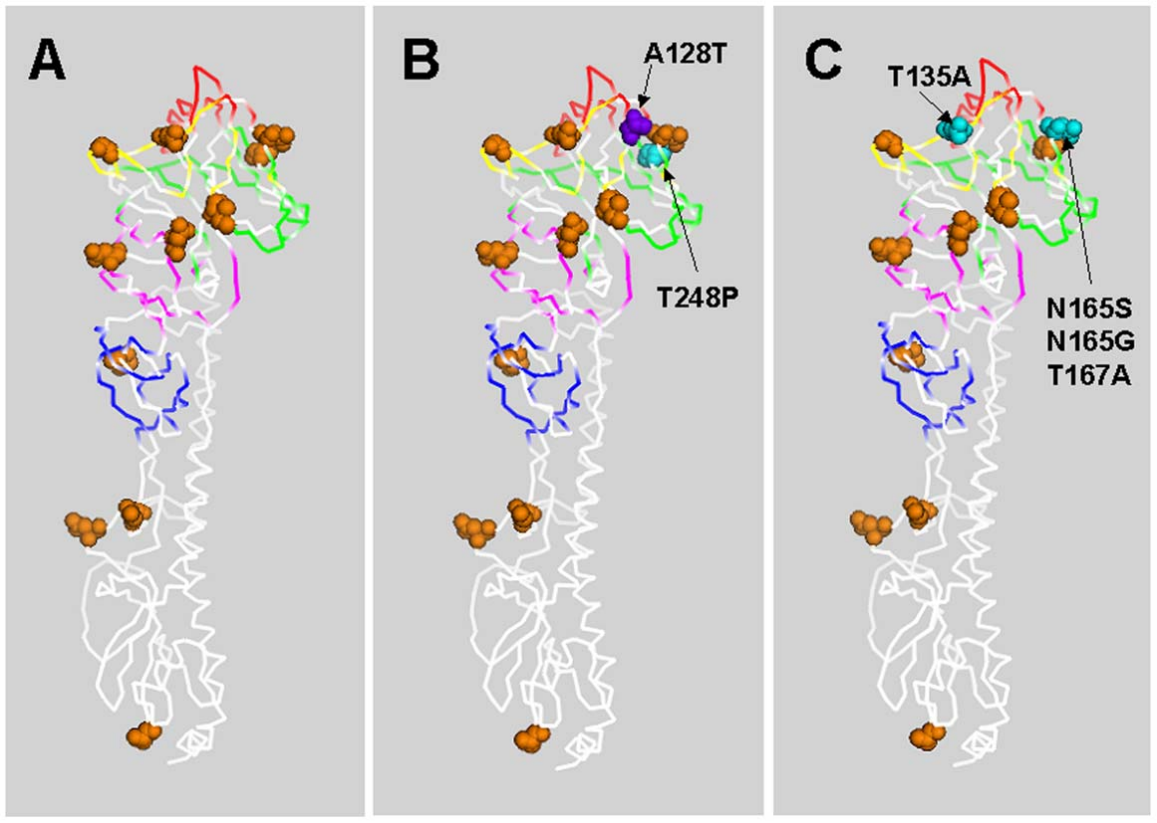
Analysis of Glycosylations. Space-filled model of HA ectodomain from related H3N2 strain X31 drawn with PyMOL using 2VIR.pdb [19]. Panel A shows the H3N2 glycosylation sites of the wild type Wyoming/2003, Panel B shows glycosylation site changes in the naïve Guinea pigs, and Panel C shows glycosylation site changes in the immunized Guinea pigs. Epitopes A, B, C, D, E are shown colorized in yellow, red, blue, green and pink, respectively. Receptor binding site is shown as mesh. Residues at which glycosylation sites were either lost during the evolution of the virus in Guinea pigs are shown in cyan, or sites that were added in purple.

Changes in glycosylation are associated with biological properties such as the folding and assembly of trimeric HA macromolecules, recognition of cell receptors, and evasion of host immunity. The number of putative glycosylation residues in circulating H3N2 influenza virus isolates has increased with time from seven glycosylations in the 1968 Aichi strain to eleven in the 2003 Wyoming strain and to thirteen in the 2007 Brisbane strain. Table 3 shows the number of changes to variants that affected the putative_glycosylation status of the HA protein with the identification of the site of the mutation. Of the variants isolated from naïve Guinea pigs, one each lost and gained a putative_glycosylation site. In contrast, five variants from immunized animals lost, and none gained, a putative glycosylation site. It is interesting to note that putative glycosylation sites at 248, 128, and 135 glycosylation sites are used in the Wyoming strain, but not its predecessors Aichi or X31 from 1968. In Figure 4, the sites of putative glycosylation change found in the progeny variants from immunized animals are identified in color and with arrows. As expected, the majority of the changes that could affect glycosylation status are seen on the surface of the HA molecule and in epitopes.

**Table 3:** Analysis of amino acid substitutions in HA variants.

Animal groups	Immune status	Loss of glycosylation	Gain of glycosylation
Group 1	Naïve	1 (T248P)	1 (A128T)
Group 2	immunized	5 (N165S, N165G, T167A, T167A, T135A)	None

## Discussion

Influenza viruses must escape from the immune pressure by antigenic variation to propagate over time in the population of host targets. The high mutation rate of RNA viruses appears to provide a ready supply of variants, some of which may have replicative advantages in the face of host immunity [21], [22]. Analysis of naturally occurring variants isolated from infected humans and other species has led to the identification of “hot spots” of mutation that are hypothesized to define amino acid residues associated with antigenic epitopes [21], [23], [24], [25]. The most variable of the residues are predicted to reside within the dominant epitopes and these epitopes, for the most part, have been confirmed by immunological studies and the data within these experiments. Multiple strategies have been employed to characterize immunodominant epitopes in viruses [11]. But due to the complexity of characterizing the evolution of influenza virus through infection of human hosts, there is great value in studying experimental infections in animal models [24]. In the current study, we surveyed progeny virus directly from Guinea pigs infected with influenza virus by sequencing multiple HA genes without passage through cell culture passage. Rather than characterizing the most common sequence at each residue by bulk sequencing of mixed templates, we sampled individual progeny to uncover specific variants. Because the majority of natural variations observed during passage of influenza virus through the human population reside in the globular head of the HA, the study was limited to analysis of the HA-1 subunit The methodology used to characterize the variants (sequencing small libraries of cDNA amplicons) does distinguish between different fitness levels of progeny variants. Although it is possible that some of the mutations may have reduced replicative fitness, the “hot spots” observed are in regions of high genetic and structural flexibility known to tolerate variations and mutations.

As expected, infection of HA-immunized Guinea pigs resulted in the selection of a higher percentage of progeny strains containing variations in the HA protein sequences as compared to unimmunized animals (24.3% vs. 6.0%, Table 1). Of the 396 residues of the HA-1 subunit analyzed during these studies, 265 amino acids, or 67%, are outside and 131 residues, or 33%, are inside epitopes. 61.5% of the mutations characterized from the nasal washes of naïve animals were located in amino acid residues outside previously defined epitopes. Thus, in the absence of immune pressure, the location of the variations resembled a random distribution. In contrast, 63.5% of the mutations found in immunized animals were within epitopes; their frequency was approximately twice the rate expected (33%) for a random distribution (data not shown).

**Figure 5:**
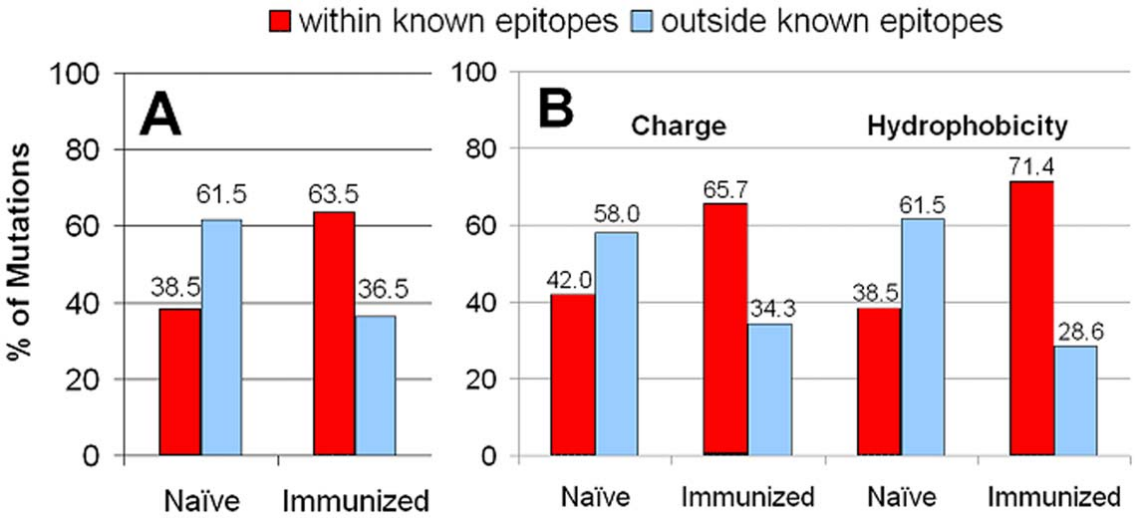
Relationships between frequency of HA mutations, epitopes, and charged or hydrophobic amino acids. Bars show frequency of mutations at each amino acid observed in variants isolated from nasal washes of influenza virus infected Guinea pigs. Frequency calculated by dividing number of mutations at a given residue with the total number of variants observed. Panel A: variants from naïve Guinea pigs, Panel B: variants from HA-immunized animals.

In addition to an analysis of the frequency and antigenic location of mutations, the physical properties of the amino acid substitutions were analyzed. Amino acids that change local electrostatic charge or hydrophobicity can have major effects on the recognition of epitopes by existing antibodies. Mutations that change these two properties can facilitate evolutionary escape and the formation of new strains of virus. In naïve animals, approximately 42% of amino acid substitutions that resulted in a change in electrostatic charge were in epitopes while 65.7% of the charge changes in progeny virus from immunized Guinea pigs were in epitopes. Similarly, 37.5% of the mutations changing the hydrophobic nature of the residue were in epitopes in naïve animals, and 71.4% were in the epitopes of immunized Guinea pigs (Figure 5). The pattern of influenza evolution in Guinea pigs correlated with mutations observed in H3N2 strains sequenced from human samples. Much of the evolution of influenza in both species involved substitutions of charged or hydrophobic amino acid residues or mutations that led to changes in glycosylation profile [18]. Influenza virus undergoing escape evolution in Guinea pigs also showed a tendency to substitute the amino acids in the epitope regions, which was also observed in human influenza [6], [27]. Consequently, the evolution of the virus in Guinea pigs represents a valuable model system for helping us to understand the escape evolution of human influenza virus. These results conformed nicely to recent analyses of sequences from human clinical isolates of H3N2 during its evolution from 1968 [18].

The HA has accumulated glycosylations during the evolution of H3N2. Over the past 40 years, the number of glycosylation signals has increased from 7 (Aichi/68) to 13 (Brisbane/2007) and appears to be one method by which a virus can mask its epitopes from immune surveillance [28], [29]. In addition, some studies have associated increasing glycosylations with reduced virulence [29], [30] while others associated the loss of glycosylations with exposure of new receptor binding sites or new decoy epitopes [31]. Michele et al (1993) reported the role of glycosylation in the control of host range and suggest that oligosaccharides on the tip of the HA are important to the survival of H1 viruses in humans but not in ducks or swine [32]. In the Guinea pig study, one of the strains from the naïve group gained a glycosylation and one lost a glycosylation (Table 3). In contrast, five of the strains from the immunized group lost glycosylation sites. Glycosylations were lost at one site each in the A, B, and D epitopes (T135, N165, and T167, respectively). Although the reasons for, and consequences of, changes to glycosylation density during evolution in humans remains unclear, it appears that the reduction in glycosylations in the major epitopes have facilitated escape from immune pressure or increased the rate of replication.

The analysis of mutation frequency identified four hot-spots of mutation regions in this study (Figure 4). It is possible that these sites of high variation enable the virus to stimulate strain-specific immunity. As such, they could be regarded as immunological “decoys” to prevent the host from developing durable immunity against multiple strains of H3N2 virus. During viral infection, the immune system appears to react preferentially against immunodominant epitopes which, in the case of influenza HA, are composed of genetically variable amino acids. It appears that influenza, and many other pathogens, have evolved to take advantage of weaknesses in the immune system caused by antigenic hierarchy. Recent studies have shown that rarely occurring antibodies can be isolated or produced that recognize highly conserved epitopes in the stalk regions of HA (HA2) [42]. We hypothesize that influenza, and other viruses that exist as antigenic swarms, have evolved such that the many of the sites that are indispensable for replication and require a high degree of genetic conservation are also poorly immunogenic. Thus, the stimulation of immunity to variable regions allows the virus to antigenically escape and, thus, the host is susceptible to antigenic variants in succeeding exposures. The observations presented herein are consistent with the results of David et al, who proposed that the evolvability of virus is a selectable trait [33].

For reasons still unexplained, the host immune system responds to many pathogens, such as influenza, by mounting a relatively oligoclonal response that is focused upon a small number of immunodominant epitopes [34]. We propose that influenza viruses evolved to take advantage of host immunological hierarchy by placing immunodominant epitopes within regions, such as unconstrained loop structures, that can accept a high degree of diversity without major decreases in replicative fitness. Thus, through relatively modest mutations in a very small number of amino acid residues, influenza can antigenically escape immune pressure. If the host could mount a truly polyclonal immune response that recognizes a large number of sites in the HA, including those that are normally sub-dominant, perhaps influenza would not elicit strain-specific immunity.

Several strategies are in development to counteract strain-specific immunity for the development of broadly cross-reactive vaccines. Vaccines targeting more conserved proteins, such as the M2, or those containing improved adjuvants have produced a range of results, including some that are highly promising [26], [35], [36]. In addition, a rational antigen design strategy designated, the Immune Refocusing Technology, represents a highly promising approach to the development of broadly protective vaccines [7], [37], [38]. In applications with influenza, Tobin et al have antigenically dampened multiple immunodominant HA epitopes of the A/Wyoming/2003 strain to engineer an initial panel of vaccine candidates that stimulate enhanced cross-reactive antiviral responses to other H3N2 strains [7]. The hot spots of mutation, and the sites that lost glycosylations, identified in the present study are potential targets of Immune Refocusing for the development of improved vaccines.

### Conclusion

HA specific immunity plays a key role in H3N2 virus evolution. The fixation of mutations of H3N2 virus in both naïve and immune animal appeared to be non-random events as immune pressure can lead to mutations accumulating in dominant epitopes. We found that the mutations driven by immunity are frequently associated with charge, hydrophobicity, and lost glycosylations. The results of the present study increase our understanding of the direction of evolution in influenza and may provide useful insights for the selection of strains to be included in the seasonal vaccine.

## Materials and Methods

### Ethics statement

All proposed animal work was reviewed by the Biological Mimetics, Inc. Institutional Animal Care and Use Committee. I-ACUC approval was obtained prior to initiation of animal work. All animal work was conducted in accordance to national and international guidelines to minimize discomfort to animals.

### Cells and viruses

MDCK cells (ATCC # CCL-34) were used for virus propagation and titration, and were maintained in DMEM supplemented with 7% fetal bovine serum (FBS). A sample of Wyoming/03 X PR8 X161B virus was obtained from the Centers for Disease Control and Prevention (CDC). The stock is a biological re-assortment of A/Wyoming/03/2003 (WYM) and A/Puerto Rico/8/1934 (PR8) and was prepared for the development of vaccine preparations by double infection of embryonated hens' eggs following antibody selection methods [39]. Sequence analysis of multiple plaques from the CDC stock demonstrated that the material was heterogenous in HA gene sequence. In order to prepare a homogeneous virus for infection of Guinea pigs, several parallel plaque-purified isolates were propagated, sequenced, and titered. The isolate used in the current study, Wy-B4, is a plaque-purified isolate from Wyoming/03 X PR8 X161B and carries an HA gene segment identical to the GenBank sequence (accession number AAT08000).

### Plaque assays

Plaque assays were used for purification of virus isolates and quantitation of virus propagated both *in vitro* and in the Guinea pig. Briefly, 6-well plates were seeded at 50% confluence with MDCK cells. The following day, serial dilutions of influenza virus were applied in 1-mL volumes for 1 hour. The monolayers were washed with PBS and overlain with a solution of 0.8% agarose in EMEM supplemented with 3.5% FBS and 0.1% neutral red. Clear plaques were visible against the stained cells after 3–4 days incubation at 37°C. The Wy-B4 isolate was purified from a well-separated plaque and propagated on fresh cultures of MDCK. Virus stocks were titered in MDCK cells prior to use in animals.

### Guinea pig Immunizations

Guinea pigs were immunized with recombinant HA ectodomain protein derived from the Wyoming/03/2003 virus. Briefly, the HA gene from the Wyoming virus was truncated at the transmembrane domain and cloned into the insect cell expression vector, pMT-BiP (Invitrogen). Induction of stably transfected S2 drosophila cells led to the soluble expression of ectodomain proteins that appear to assemble into trimeric structures by small angle X-ray scatter (SAXS analysis, data not shown). The recombinant protein was purified by sequential His-tag selection on metal activated sepharose, lentil lectin agarose, and DE-53 anion exchange resins. A mock preparation of antigen was prepared by the same manner using empty pMT-BIP vector. Harlan Sprague Guinea pigs, 6–8 weeks of age, were obtained (Harlan-Spraque-Dowley, Inc.) and divided into two groups. Group 1 was given the mock antigen and served as a negative control for the experiment. Group 2 was given the HA recombinant ecto domain protein. The purified protein was compounded with Complete Freund's Adjuvant for initial immunizations and Incomplete Freund's Adjuvant for boosts at weeks 3, 5, and 10. Serum samples were taken periodically throughout the study for serological analysis.

### Guinea pig infection and nasal wash collection

All of the animal work was performed at BioCon, Inc. (Rockville, MD) under AAALAC guidelines and I-ACUC- and ACURO-approved animal study protocol documents. Approval of BMI I-ACUC study protocols preceded the initiation of any animal work. Lightly anesthetized Guinea pigs, naïve or immunized, were inoculated intranasally with approximately 10^4^ plaque forming units (PFU) of the Wy-B4 isolate in 0.3 mL volumes of sterile saline. Virus progeny were sampled by nasal irrigation of anesthetized Guinea pigs using 0.5 mL PBS supplemented with 100 ug/mL penicillin, 100 ug/mL streptomycin and 0.3% bovine serum albumin [8]. Nasal washes were clarified of debris by centrifugation at 1,600×*g* for 10 min and stored at −80°C prior to analysis. Where noted, Guinea pigs were re-infected after a 6-week recovery period using the same dose and route.

### ELISA Analysis of Guinea Pig Sera

Immune responses were monitored by ELISA method. Briefly, Nunc Maxisorb flat-bottom 96-well plates were coated overnight with full-length A/Wyoming/03/2003 HA protein (Protein Science Corporation) at a concentration of 1.5 micrograms in 0.1 mL of PBS per well. The plates were washed in PBS containing 0.1% Tween-20 (PBS-T) and then nonspecific binding blocked with 0.2 mL/well of 10% nonfat dried milk in PBS for 2 h at 37°C. Serum samples were serially diluted in 1% milk solution and 0.1 mL aliquots were tested for binding to antigen in triplicate wells. Plates were incubated for 1 h at 37°C and then washed again using PBS-T. The plates were probed with a peroxidase-conjugated goat anti-Guinea pig total IgG antibody (Kirkegaard & Perry Laboratories, Inc., Gaithersburg, MD, KPL, 1∶1000) for 1 h and then washed again with PBS-T. Bound conjugates were quantitated by the addition of 0.1 mL tetramethylbenzidine (TMB) substrate (KPL) for 90 sec, immediately followed by an equal volume of 0.1 N sulfuric acid. Plates were read at 450 nm. The triplicate well values were used to calculate mean average values. Values from antigen-coated wells reacted with secondary antibody, but not test sera, were used as plate background values and were subtracted from absorbance values in test wells. ELISA extinction titers were calculated as the maximum serum dilutions that resulted in a signal that exceeded a value that was three times plate background (approximately 0.15 OD units). Mean values with error bars equal to one standard deviation of the triplicate were graphed as a function of time over the course of the study and, due to their small magnitudes, may not be visible on the graphs.

### Virus Microneutralization (VN)

Detection of serum neutralizing antibodies was determined by a standard method [40], with a few modifications. Briefly, in a 96-well plate, two-fold dilutions (10 µl volume) of RDE-treated sera were prepared over a concentration range of 1∶20–1∶2560 in assay diluent (1% bovine serum albumen in PBS containing penicillin and streptomycin). The plate was incubated with A/Wyoming virus (100 TCID_50_) for 2 hrs at 37°C. MDCK cells (3×10^4^) were added and incubated 18 hrs at 37°C, 5% CO_2_. The cells were fixed with 80% acetone, blocked with assay diluent containing 1% Tween-20, and probed for nucleoprotein (NP) with mouse anti-influenza antibodies (1∶4000 final concentration of 1∶1 MAB8258 and MAB8257, Millipore, Billerica, MA). After extensive washing, goat anti-mouse IgG HRP (1∶2000, Jackson Labs, West Grove, PA) was added for 1 hr at RT followed by color development by quantification using tetramethylbenzidine (R&D System, Minneapolis, MN). Absorbance was read at 450 nm.

### Hemagglutination Inhibition Assay (HI)

A standard HI assay was performed in blinded fashion to assess antiviral antibodies [41]. Prior to assay, serum samples were treated with Receptor Destroying Enzyme (RDE, Denka Seiken CO LTD., Tokyo, Japan) overnight at 37°C followed by heat inactivation for 1 hour at 56°C. Two-fold dilutions of serum samples were mixed with A/Wyoming/03/2003 virus (at a concentration of 4 hemagglutination units per well) and incubated for 15 min at room temperature. 0.05 mL of a 0.5% suspension of chicken red blood cells was added and hemagglutination was assessed after 1 h, as described.

### Sequences analysis

Viral RNAs were extracted from nasal wash and tissue culture samples using the RNA Easy Kit (Qiagen). cDNAs were synthesized using Superscript reverse transcriptase (Invitrogen) and the following positive strand primer: 5′-ATGAAGACTATCATTGCTTTAAG-3′. HA cDNA fragments were amplified by PCR using the above oligonucleotide with the negative strand primer: 5′-CGCGATTGCGCCAAATATGCC-3′ and a maximum number of 18 cycles. Pfu polymerase (Stratagene) with proofreading activity was used to minimize mutations introduced in the PCR amplificaiton process. The 1056-nt fragment of gene segment 4 containing the HA-1 coding region for was cloned into the TOPO TA vector (Invitrogen) and independent colonies were selected for DNA sequencing. DNA sequences of the HA1 fragments were compared to the original sequence of the Wyoming/03/2003 HA1 gene fragment (Genbank accession number AAT08000).

### Data analysis

A Chi-squared statistical analysis of homogeneity was used to test the hypothesis that two percentages of mutations are equal between different test groups. The formula used for the test is:
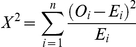
where n = number of samples in set, A = the probability (p) assigned to the null hypothesis that there is no difference between the two sets of data. As a test of statistical significance, a confidence level of 95%, p<0.05, was applied.

### Ethics Statement

Animal studies were performed within the guidelines of United States animal study protocol requirements. Guinea pig studies were performed at BIOCON, Rockville, MD. BIOCON has an approved Animal Welfare Assurance Statement on file with the Office of Laboratory Animal Welfare (OLAW, #A3267-01); is accredited by the Association for the Assessment and Accreditation of Laboratory Animal Care International (AAALAC, #000529); and is registered with the U.S. Department of Agriculture (USDA, #51-R-032). Prior to starting animal studies, all protocols were approved by Institutional Animal Care and Use Committees at both BioCon, Inc. (Protocol number A0814-07B) and Biological Mimetics, Inc. (Protocol number ACUC-051807).

## Supporting Information

Table S1 **List of variant progeny sequences from naïve Guinea pigs.**
(DOC)

Table S2 **List of variant progeny sequences from immunized Guinea pigs.**
(DOC)
