# Supplementary material for: Evolution of H3N2 Influenza Virus in a Guinea Pig Model
Source: PLoS One. 2011 Jul 22;6(7):e20130. doi: 10.1371/journal.pone.0020130 (PMC3142111; doi:10.1371/journal.pone.0020130)
Supplement: Table S1 — List of variant progeny sequences from naïve Guinea pigs. (DOC) [file pone.0020130.s001.doc]

| Table S1: List of nonsynonymous mutations observed in progeny strains from naïve Guinea pigs | | | | | | | | |
| --- | --- | --- | --- | --- | --- | --- | --- | --- |
| Strain | Variant | Epitope | Strain | Variant | Epitope | Strain | Variant | Epitope |
| 1 | V130D | A | 10 | L154M | O | 22 | K173E | D |
| 1 | A191H | O | 11 | G49D | O | 23 | G342S | O |
| 1 | S193T | B | 12 | G181A | O | 23 | N424D | O |
| 2 | C52R | O | 13 | I260T | E | 24 | T248P | D |
| 3 | L151S | O | 14 | L164V | O | 25 | G132D | A |
| 4 | K264R | O | 15 | Q44H | C | 26 | Q327K | O |
| 4 | K140R | A | 16 | A39V | O | 27 | H56Y | O |
| 5 | V112G | O | 17 | H184P | O | 27 | F146S | A |
| 6 | Q132H | A | 18 | L316S | O | 28 | I25V | O |
| 7 | Q132H | A | 19 | L71S | O | 28 | E123G | O |
| 7 | N126Y | A | 20 | D271G | O | 29 | K276Q | C |
| 8 | C14R | O | 21 | L384S | O | 30 | G50R | C |
| 9 | A128T | B | 21 | V20A | O | 31 | Q211K | O |
