# Supplementary material for: Evolution of H3N2 Influenza Virus in a Guinea Pig Model
Source: PLoS One. 2011 Jul 22;6(7):e20130. doi: 10.1371/journal.pone.0020130 (PMC3142111; doi:10.1371/journal.pone.0020130)
Supplement: Table S2 — List of variant progeny sequences from immunized Guinea pigs. (DOC) [file pone.0020130.s002.doc]

| **Table S2: List of nonsynonymous mutations observed in progeny strains from Immunized Guinea pigs** | | | | | | | | |
| --- | --- | --- | --- | --- | --- | --- | --- | --- |
| Strain | Variant | Epitope | Strain | Variant | Epitope | Strain | Variant | Epitope |
| 1 | V112I | O | 21 | N152D | A | 35 | N126D | A |
| 2 | Q197R | B | 22 | G129E | B | 36 | T135A | A |
| 3 | Q211K | O | 23 | N125D | O | 36 | K145R | A |
| 4 | D79G | O | 24 | C52R, | O | 37 | K259E | O |
| 5 | G129E | B | 24 | K92E | E | 38 | N250D | O |
| 6 | N165S | B | 24 | V182A | D | 39 | D77N | O |
| 7 | C76R | O | 24 | R228G | D | 40 | Q75R | E |
| 8 | Q211K | O | 25 | D53V | C | 41 | E172G | D |
| 9 | T167A | D | 26 | E62K | E | 41 | S189G | B |
| 10 | I58V | O | 27 | P74L | O | 42 | P169L | O |
| 11 | E41G | O | 28 | T65A | O | 43 | S209G | D |
| 11 | D188N | B | 29 | P74L | O | 44 | N171S | D |
| 12 | T117A | D | 30 | I58T | O | 45 | I226V | D |
| 13 | S45G | C | 31 | E123K | O | 45 | R229G | D |
| 14 | Y195H | O | 32 | L118P, | O | 46 | P169L | O |
| 15 | T167A | B | 32 | R229G | D | 47 | V43I | O |
| 16 | G50E | C | 32 | G240E | D | 48 | K92R | E |
| 17 | L194P | B | 33 | D190G | B | 49 | A163V | B |
| 18 | G129E | B | 33 | R142K | A | 50 | R299G | C |
| 19 | G129E | B | 33 | I252V | O | 51 | D173G | D |
| 20 | N152D | A | 34 | Y161H | O | 52 | E165G | B |
